# Revisiting nephrin signaling and its specialized effects on the uniquely adaptable podocyte

**DOI:** 10.1042/BCJ20230234

**Published:** 2025-06-02

**Authors:** Casey R. Williamson, Una V. Pantic, Alice Y. Wang, Nina Jones

**Affiliations:** 1 Department of Molecular and Cellular Biology, University of Guelph, Guelph, Ontario, Canada

**Keywords:** cell signaling, chronic kidney disease, focal adhesion, nephrin, podocytes

## Abstract

Nephrin is a transmembrane Ig-like domain-containing protein that serves as a central structural and signaling scaffold in kidney filtration. First identified in 1998 as mutated in congenital nephrotic syndrome, the recent identification of nephrin autoantibodies in acquired kidney diseases has sparked renewed interest in nephrin biology. In specialized cells known as podocytes, nephrin helps establish and maintain the slit diaphragm (SD), a unique cell–cell junction formed between interdigitating cell projections known as foot processes (FPs). Together, the SD and FP are among the first stages of renal filtration, where they are subject to numerous biochemical and mechanical stressors. Although podocytes are highly adapted to this environment, over time and with injury, this elevated strain can lead to pathological structural changes, detachment, and proteinuria. As such, the complex set of signaling mechanisms provided by nephrin are essential for controlling podocyte adaptability. Herein, we provide a thorough and up-to-date review on nephrin signaling, including a focus on cross-talk between nephrin interactors and signaling regions across podocytes. We first highlight new findings regarding podocyte structure and function, followed by an emphasis on why nephrin is among the most critical proteins for maintaining these features. We then detail a comprehensive list of known nephrin interactors and describe several of their effects, including calcium regulation, cell survival, cell polarity, phase separation-mediated actin reorganization, and SD–focal adhesion dynamics. Collectively, our emerging understanding of the broader cellular context of nephrin signaling provides important insight for clinical strategies to mitigate podocyte injury and kidney disease progression.

## Introduction

Nephrin is a major structural and scaffolding protein carefully confined within the podocyte membrane, and its multiple signaling mechanisms are essential throughout life for controlling podocyte integrity and kidney function. Within the kidney, podocytes are highly specialized cells that form a major layer of the glomerular filtration barrier (GFB) and help control the earliest stage of blood filtration. With approximately 180 liters of plasma passing through this filter every day [1], this unique cellular setting leads to substantial mechanical, chemical, and biochemical stressors that are continuously placed against podocytes. Although signaling strategies through proteins such as nephrin have been adapted by podocytes to resist these stressors, pathological challenges can provoke drastic and potentially life-threatening podocyte damage. Such injury is often irreversible, as podocytes have a limited capacity for regeneration, restricting most clinical interventions at late stages to dialysis or kidney transplantation [2]. Consistent with their vulnerability, numerous mutations in podocyte-affecting genes including nephrin have been associated with kidney dysfunction, and treating these genetic conditions is now a focus for modern clinical strategies [3,4]. Given the central and critical role of podocytes in kidney health, investigating the adaptive mechanisms that maintain their structural and functional integrity is a research priority.

To this end, great progress has been made to further understand the inherent resilience of podocytes and, specifically, their ability to continually adapt to microenvironment stress. Several advances relate to podocyte metabolism [5–7], cytoarchitecture [8–10], and regeneration [11,12], while other research efforts have helped explain the complex signaling mechanisms originating from podocyte foot processes (FPs), where nephrin is specifically localized. These interdigitating cellular projections perform vital podocyte functions that include FP-matrix adhesion to the underlying glomerular basement membrane (GBM) [13] and FP-FP (cell–cell) adhesion via their modified adherens junction, the slit diaphragm (SD) [14]. Nephrin is a defining component of the SD, and it contributes to actin-regulatory mechanisms in FPs [15,16] through a diverse group of known binding partners, many of which were elucidated shortly after its discovery by Kestilä et al. in 1998 [17]. While this initial speed in discovering nephrin interactors appears to have slowed in the last decade, recent studies have echoed nephrin’s importance in podocyte and kidney function [18–20]. However, most signaling abilities downstream of nephrin have not been spatiotemporally contextualized in the dynamic microenvironment of podocyte FPs. In this review, we therefore aim to provide an important update on nephrin and describe how its precise localization within FPs, along with its distinct signaling features, is implicated in adaptive functions. Notably, we particularly focus on nephrin’s cross-talk between the SD and adhesion complexes, which is likely a central function for podocyte resiliency.

### The unique cellular context of podocytes

#### Nephrons and the GFB

**Figure 1:**
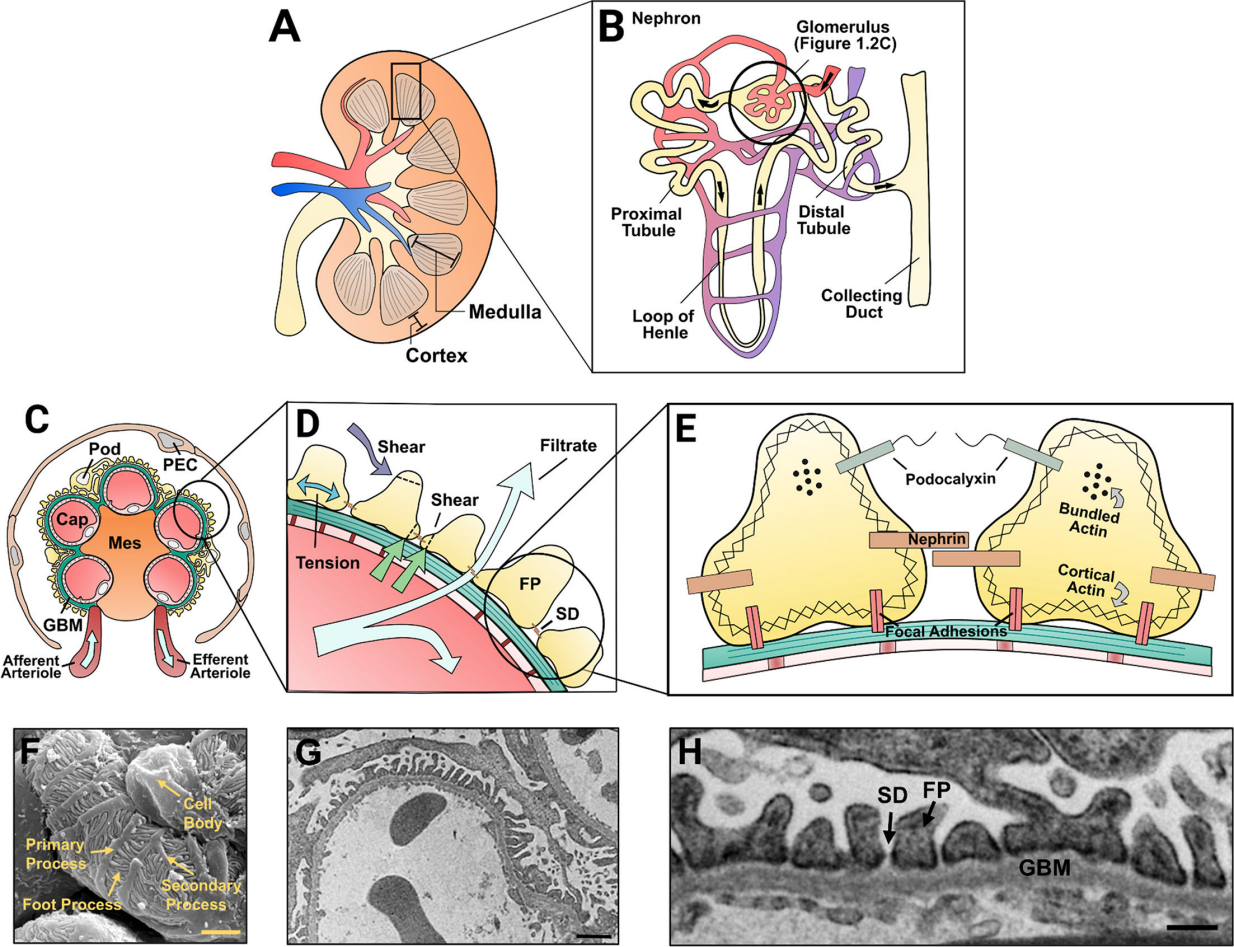
Podocyte localization, structure, and microenvironment.

First, defining nephrin’s role in podocyte adaptability requires consideration of the podocyte microenvironment within the overall nephron. Nephrons are the functional units of the kidney and are primary elements in the renal cortex and medulla (Figure 1A and B), where the total nephron count can vary largely between individuals with reports ranging from 200,000 to 2.7 million per kidney [21]. During the initial stage of filtration, blood flow enters the glomerulus via the afferent arteriole, and filtrate is first created by the passage of waste products through the GFB. Here, filtrate must traverse three layers of the GFB: the innermost fenestrated endothelial cells/capillaries, the middle GBM, and the outermost podocytes (Figure 1C), where filtrate passes between FPs and through the nephrin-bridged SD (Figure 1D) [22,23]. The GFB is also supported by an inner core of mesangial cells which help with secretion of GBM and mesangial matrix, regulation of inflammation, and matrix phagocytosis (Figure 1C) [24]. The GFB is both size-selective via endothelial fenestrations, GBM fibers, SD width, and SD membrane proteins and charge-selective by the negatively charged endothelial glycocalyx, GBM heparan sulfate proteoglycans (HSPGs), and podocyte glycocalyx [22,23,25]. Large, negatively charged plasma components (importantly albumin) are therefore retained at high efficiencies in the capillaries, exiting the glomerulus through the efferent arteriole. In contrast, water and smaller solutes can passively cross the GFB to enter a tubular system that precisely regulates their reabsorption into the blood. This system sequentially consists of the proximal convoluted tubule, loop of Henle, distal convoluted tubule, and collecting duct (Figure 1B), each having distinct reabsorption and secretion abilities to regulate overall excretion. Furthermore, the macula densa of the distal convoluted tubule provides important feedback that controls glomerular filtration rate (GFR) in relation to filtrate concentrations. In this way, the glomerulus and tubules form a highly co-ordinated unit that utilizes active signaling for effective excretion homeostasis.

(**A**) Illustration and structure of a coronally-sectioned kidney. Nephrons are primary components of the medulla (tubules and collecting ducts) and cortex (tubules and glomeruli). (**B**) Structure of the nephron. Blood flow enters glomerular capillaries, and filtrate composition is adjusted through reabsorption/secretion while traveling through the proximal tubule, descending/ascending limbs of the loop of Henle, distal tubule, and collecting duct. Circle indicates glomerulus illustrated in (**C**). (**C**) Structure of the glomerulus. Podocytes (Pod) adhere to the glomerular basement membrane (GBM), which surround fenestrated capillaries (Cap) and are supported by the inner mesangium (Mes). Parietal epithelial cells (PEC) line the Bowman’s capsule that surrounds the glomerulus. (**D**) Diagram of filtrate flow through the glomerular filtration barrier, and the shear and tension stress forces placed on podocytes. Individual podocyte foot processes (FPs) are shown, with the slit diaphragm (SD) between them. (**E**) Image of two FPs, with *trans*-interaction of nephrin’s extracellular region at the SD. Podocalyxin, nephrin, and focal adhesions are primary components of the apical, basolateral, and basal domains, respectively. Actin is distributed across the FP, and both cortical and bundled longitudinal fibers are shown. (**F**) Scanning electron microscopy (SEM) image of a podocyte surrounding the GBM and capillary. Podocytes project primary processes from their cell body, which further divide into secondary and tertiary FPs. (**G-H**) Transmission electron microscopy (TEM) images of the glomerular filtration barrier, at lower (**G**) and higher (**H**) magnifications. Scale bars in F, G, and H are 2 µm, 1 µm, and 250 nm, respectively.

#### The architecture and subcellular regions of podocytes

In light of the fluctuating signals from nephron feedback and system-wide homeostasis, it is critical for podocytes to maintain specialized structural features. Specifically, podocytes have an elaborate cytoskeletal architecture that is acquired through terminal differentiation, which greatly limits their mitotic and regenerative ability [26]. This cell state allows podocytes to wrap around the glomerular capillaries with highly arborized projections that securely adhere to the GBM (Figure 1F). Podocyte nuclei and cell bodies are suspended in the Bowman’s space, typically nestled in the grooves between capillary loops. From this space, they project primary and secondary processes (collectively termed major processes) formed by microtubules, intermediate filaments, and a contractile actomyosin network of non-muscle myosin-IIa (NM-IIa) [27,28]. Many of these major processes then extend parallel ridge-like prominences (RLPs) on their basal surface that attach to the GBM [9]. Here, actin-rich FPs project from the RLPs and interdigitate with FPs from neighboring podocytes, forming the SD at their cell–cell contacts and GBM adhesions on their basal surface [27]. Finally, other forms of projections, including microprojections/microvilli and tunneling nanotubes, can be observed on podocyte apical surfaces [29–31]. Throughout the above structures, podocytes also possess many features involved in membrane/protein trafficking, such as prominent rough/smooth endoplasmic reticula and Golgi apparatus at their cell body and abundant endo- and exocytic vesicles and lysosomes across their structure [32,33].

To facilitate cell–cell and cell–matrix adhesions, podocyte FPs possess prominently polarized basal, apical, and basolateral domains. The basal domain provides dynamic GBM adherence and contains elements like α3β1, α2β1, and αVβ3 integrin-based focal adhesions (FAs) (Figure 1E), along with CD151, DDR1, dystroglycan, type XVII collagen, transmembrane HSPGs glypican-1, and syndecan-1 and -4 [13,34,35]. Facing the Bowman’s space, the FP apical domain contains glycoproteins such as podocalyxin (Figure 1E), GLEPP1, and podoendin, where their negatively charged glycocalyx is hypothesized to help with charge-selective filtration and interspacing of FPs [36]. Between these apical and basal domains, FPs have characteristically curved basolateral domains, where the essential nephrin-based SD signaling platform is localized. The nature of cell–cell adhesion at the SD can be described as a modified adherens junction as it contains P-cadherin, protocadherin FAT1, α/β/γ- and p120-catenins, and ZO-1 [14,37]. Among these proteins, the SD contains two essential layers of cell–cell adhesion, where nephrin forms the wider layer of approximately 45 nm closer to the apical domain, and the related transmembrane protein NEPH1 forms a narrow layer approximately 23 nm closer to the basal domain [38,39]. The underlying basolateral membrane is also a unique ganglioside and cholesterol-rich lipid raft, and the composition of this raft is partly regulated by the scaffolding protein podocin [40,41]. Between each of these compartments, the actin-rich FP intracellular space consists of highly branched sublemmal actin and a longitudinal bundle of filamentous actin (F-actin) centered in the apical domain (Figure 1E) [42]. The basal, apical, and basolateral domains collectively control these actin networks through regulatory mechanisms including membrane proteins (e.g., nephrin), downstream adaptor proteins, actin scaffolds, Rho GTPases, guanine nucleotide exchange factors (GEFs), and GTPase-activating proteins (GAPs) [43–45]. Considering these shared signaling pathways, the proximity of the FP domains suggests that cross-talk between them could be a crucial feature in adaptive responses against stressors.

#### Mechanisms to resist mechanical stress forces

Notably, high hydrostatic and hydrodynamic capillary pressures result in podocytes being exposed to a variety of shear and tension forces (Figure 1D) [46]. Filtrate flow is a primary contributor to shearing and first occurs against the FP basolateral domain as filtrate passes through the SD. Once filtrate enters the subpodocyte space (i.e., in between all major processes) and Bowman’s space, the fluid flow toward the tubules, along with fluctuating oncotic pressures, also shears against FP apical domains, major processes, and podocyte nuclei [4,46,47]. Further, the hydrostatic pressure difference observed between capillaries and the Bowman’s space leads to two tension forces: circumferential and axial wall stresses. These tension forces act mostly on the longitudinal or transverse axes of FPs, depending on the FP’s orientation along the capillary. This effect is due to FPs being predominantly in parallel with straight capillary sections [10], suggesting that podocytes orient their tension forces along their transverse axis and along the length of SD proteins (as visualized in Figure 1D). It is proposed that this orientation could permit optimal filtrate flow, allowing SD proteins such as nephrin to act as a spring against systolic/diastolic pressure variance and to prevent clogs of partially filtered solutes [10,38,46]. It is likely that the many mechanosensors at FPs also help maintain this orientation, as podocytes express mechanosensitive ion channels (e.g., TRPC5/6, P2X_4_, and Slo1) [48–51], actin-binding proteins (e.g., synaptopodin, α-actinin-4, and cofilin) [52,53], and SD, FA, and matrix components (e.g., α-catenin, p130Cas, talin, and fibronectin) [54–57]. While nephrin’s direct role as a mechanosensor remains to be investigated, it was recently shown that nephrin clustering and phosphorylation control podocyte contractility and force transmission, as detected by MLC2 activation and force mapping microscopy, respectively [58]. Furthermore, nephrin can signal/interact with many established mechanosensitive proteins (e.g., p130Cas, TRPC6, Slo1, α-actinin-4) [59–62] and is placed in an opportune area of FPs to rapidly sense changes in glomerular filtration.

#### Maintaining dynamic and persistent adhesion

Ultimately, FPs must ensure that such variable stimuli can sustain modular levels of adhesion to prevent their detachment from the GBM. If small patches of FPs are detached, and adaptive adhesive responses are compromised, the increase in shear forces might provoke a cascade of podocyte detachment that leads to irreversible injury [63]. Detachment has also been proposed to be the major contributor to irreversible podocyte loss, as living podocytes, rather than apoptotic or necrotic podocytes, are detected in the free Bowman’s space and urine of patients with glomerulopathies [63–65]. Although limited regeneration can be carried out by cells of renin lineage (CoRLs) and parietal epithelial cells (PECs) [11,31,66], they have a slow regenerative rate that cannot recover proper glomerular podocyte density [11].

Therefore, podocytes help maintain this adherence through tight control of FAs, most notably those containing α3β1 integrin complexes [13,34]. Under various contexts, FAs can be remodeled and reformed, leading to a life cycle of FA maturity that spatiotemporally regulates motility, cell contractility, cell survival signaling, and endocytosis [67,68]. Before maturation into FAs, integrin conformations alter between inactive and higher-affinity active states, and these states can be regulated by ligands, mechanical forces, clustering, lipid dynamics, transmembrane proteins, and cytoplasmic interactors to induce bidirectional outside-in and inside-out signaling [44,67,69]. As one manner of stimulating nascent adhesion (NA) formation, the cytoplasmic tail of β1 integrin can be phosphorylated to regulate its direct interactors (e.g., talin-1, kindlin-2, tensin2, filamin A/B, and DOK1), leading to altered integrin ligand affinity [13,70–74]. Although most NAs are short-lived, mechanosensitive interactors such as talin-1 and kindlin-2 can act as a molecular clutch that engages traction forces, maturing select NAs into intermediate focal complexes, FAs, or larger comet-shaped fibrillar adhesions [67,75]. Once mature, FAs typically consist of a three-layered signaling hub that includes sublemmal signaling complexes (e.g., FAK/Paxillin/p130Cas and ILK/PINCH/Parvin), actin-tethering force transduction proteins (e.g., talin, vinculin, zyxin, and Ena/VASP), and stress fiber-associated proteins (e.g., NM-IIa, α-actinin-1/4, and synaptopodin) [13,76–80]. Like the SD, FAs also consist of adaptor proteins, kinases/phosphatases, actin nucleators, Rho GTPases, GEFs, and GAPs to guide FP adhesion [13,34,81]. In this way, GBM attachment relies on active signaling rather than static adherence, which suggests an additional co-ordination of FA dynamics by nearby signaling hubs like the SD. Indeed, several studies have highlighted the existence of SD-FA cross-talk mechanisms, including nephrin’s ability to activate FA components (e.g., Rap1, β1 integrin, FAK, paxillin, and p130Cas) [58,82,83].

#### Pathological changes to FP structure and function

In many renal pathologies, it is likely this SD-FA co-ordination would largely be compromised. Podocyte injury and resulting proteinuria are often associated with a loss of podocyte specialization, commonly in the form of FP effacement. This can be observed across a wide range of podocytopathies, including diabetic nephropathy, focal segmental glomerulosclerosis (FSGS), membranous nephropathy, and in animal models of acute injury, such as protamine sulfate, Adriamycin/doxorubicin, or lipopolysaccharide administration [4,84]. One important feature of effacement is the expansion in area between the SD and any medially placed FAs within FPs. This increased distance from the SD might alter the FA signaling profile, changing their composition in a manner that promotes further podocyte effacement. Such alterations in signaling might also be accelerated by loss of SD integrity, as many injuries are associated with down-regulated SD proteins (e.g., nephrin and podocin) and up-regulated tight junction proteins (e.g., claudin-1 and occludin) [85–88]. Concomitant with these changes, GBM thickening and sclerotic lesions are also frequently observed, which suggests that altered FA ligands (e.g., fibronectin, vitronectin, and suPAR) and mechanical shifts might also drive a loss of specialized FP architecture [89–91].

Yet, it remains unclear whether these transformed features are strictly pathogenic or if they also provide an adaptive strategy. Pathologically, one suggested effect from effacement is decreased compression of the GBM (i.e., the buttress force), increasing GBM permeability and protein leakage through the GFB [92]. In contrast, it is also suggested that effacement and tight junction formation might serve as an adaptive function against detachment from increased shear stress, while maintaining the possibility of podocyte recovery if attachment is retained [63]. In this way, effacement might have roles in podocyte resiliency, but tight control of this process could depend on efficient cross-talk between the FP domains. One candidate protein that could strongly contribute to this cross-talk is nephrin.

### The nephrin SD signaling hub

#### Nephrin as a key regulator of podocyte adaptability

In addition to its unique localization at the SD, there are several clues that suggest nephrin is an important regulator of podocyte adaptability. First, it is well-established that nephrin is strongly expressed in healthy podocytes, and several proteomic and bioinformatic-based approaches have ranked nephrin among the most abundant transmembrane SD proteins [93,94]. It should be noted that NEPH1 has been reported as an SD transmembrane protein with greater expression, having a ratio to nephrin of approximately 2:1 [38,93], although other novel and abundant SD membrane proteins continue to be discovered [94]. While NEPH1’s importance cannot be understated, a larger proportion of signaling mechanisms have been discovered downstream of nephrin, which include actin remodeling [59,95], cell survival [96,97], cell polarity [98,99], calcium signaling [100,101], vesicle trafficking [102,103], mechanosensor interaction [59,101], and FA dynamics [59,83], with nephrin still having the ability to interact with NEPH1 and other abundant SD proteins such as podocin and CD2AP [39,93,104–106]. Also unique to nephrin is its ability to undergo phase separation with other multivalent interactors (e.g., Nck adaptors and N-WASP) [107]. This ability could have distinct roles that increase adaptability of FPs, such as increasing membrane dwell time of nephrin interactors [108] or rheological changes [109], which might protect against the immense shear, tension, and clogging stressors at the SD. Additionally, nephrin’s suggested spring mechanism, having potentially elastic and foldable extracellular domains [38,110,111], might reduce tension forces toward FPs, acting as a buffer against excessive mechanotransduction during normal hemodynamic strain. Importantly, it was recently shown that nephrin phosphorylation also mediates podocyte contractility and force transmission, which was concomitant with activation of FA components FAK, paxillin, and p130Cas [58]. This contractile ability could provide an adaptive response to maintain GBM compression (i.e., buttress forces) against increased mechanical strain, thus reducing excess proteinuria and podocyte detachment. Finally, there is a critical pathological relevance to nephrin’s signaling roles, as recent studies have discovered nephrin autoantibodies in a large percentage of patients with minimal change disease (44%), FSGS (9%), or idiopathic nephrotic syndromes (52–90% in certain pediatric groups), and these autoantibodies correlated with disease outcome [18,112]. Immunization of mice with anti-nephrin antibodies was also capable of increasing nephrin phosphorylation at Y1191 (corresponding to Y1176 in humans) and the levels of nephrin interactions (e.g., ShcA) [112], highlighting the importance of further characterizing nephrin’s wide range of signaling abilities.

#### Nephrin structure and signaling partners

As a principal SD transmembrane protein, nephrin possesses two functional regions: its receptive extracellular scaffold and its signal-transducing cytoplasmic tail. As a type-1 single-pass transmembrane protein, its N-terminus is directed externally, and its C-terminus is directed towards the cytoplasm [17]. Humans only express two structurally similar protein-coding isoforms, and for simplicity, this review is in reference to the canonical human isoform (Ensembl designation *NPHS1-202*). However, many of the domains and signaling motifs of this isoform are evolutionarily conserved across many species [113].

**Figure 2:**
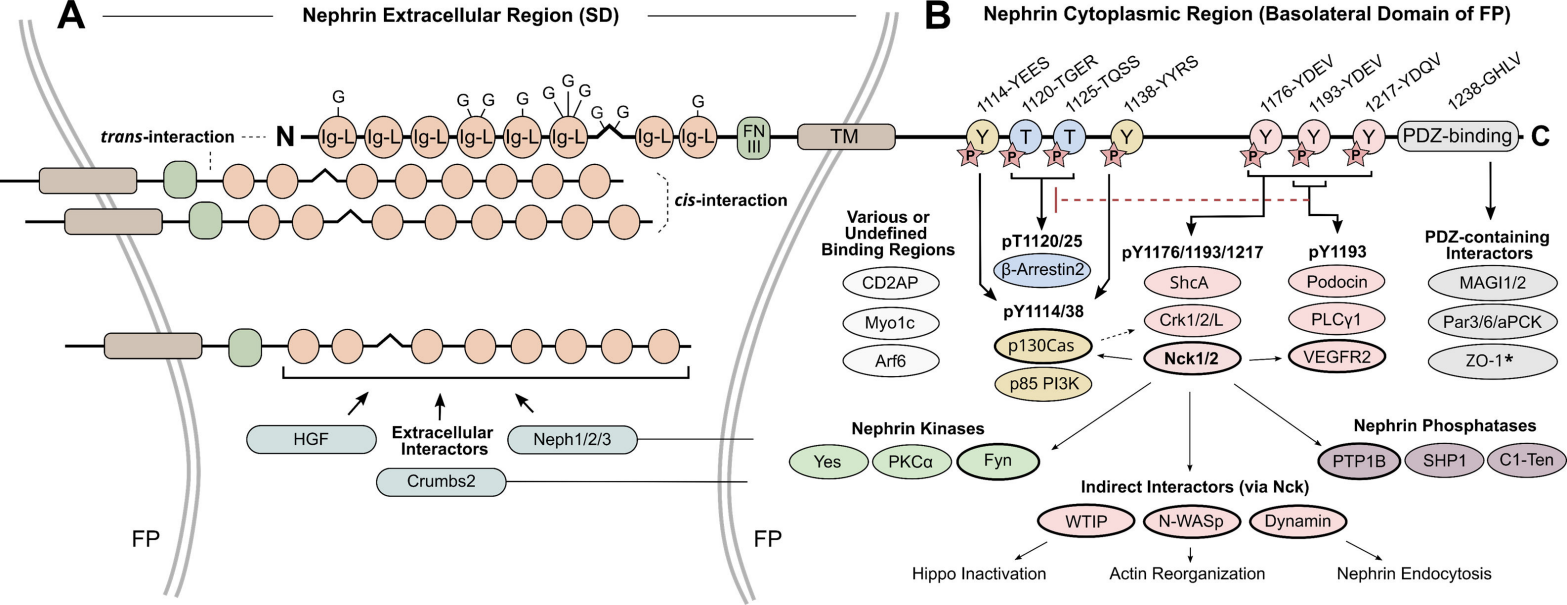
Nephrin structure at the slit diaphragm (SD) and its interactors. (**A**) The extracellular region of nephrin projects into the SD space. This region contains eight C2-type immunoglobulin-like domains (Ig-L), one fibronectin type-III domain (FNIII), and ten residues that are glycosylated (**G**). Extracellular regions of nephrin can form homophilic *cis*- and *trans*-interactions with nephrin on the same or opposing foot processes (FPs), respectively. Nephrin’s extracellular region can also be bound by secreted factors (e.g., HGF) and other SD membrane proteins (e.g., NEPH1/2/3 and Crumbs2). (**B**) The cytoplasmic tail of nephrin is situated at the basolateral domain of FPs and co-ordinates a large network of signaling processes. Conserved phosphorylated (**P**) residues and domain-binding motifs for signaling are shown with their residue number and adjacent amino acids (human sequence). Nck1/2 adaptor-binding partners are bolded as an example of diverse downstream signal transduction from nephrin. Inhibition of β-arrestin2-nephrin binding by pY1193 is indicated via dashed red line, and p130Cas contribution to Crk1/2-nephrin binding is shown with black dashed arrow. Asterisk indicates this interaction site has not been consistently observed across studies. These interactions are not comprehensive, and an extended list of interactions is given in Table 1.

The extracellular region contains six C2-type immunoglobulin-like (Ig-L) domains, a spacer region, two additional C2 Ig-L domains, and a fibronectin type-III domain (FN-III) (Figure 2A) [17]. Across these Ig-L domains, nephrin is glycosylated on ten asparagine residues, increasing its unmodified molecular weight of 135 kDa to a variable size between 150–180 kDa [114]. Like podocalyxin, these glycosylations contain sialic acid and might confer additional negative charge to the GFB [114]. Between FPs, nephrin’s extracellular region can form *cis*- and *trans*-homophilic interactions with nephrin from the same or opposing FP, and a disulfide crosslinked zipper pattern of this interaction was historically suggested to create a size-selective filter against plasma constituents [115,116]. However, the degree of *trans*-interactions forming this zipper pattern is inconsistent [38,117], and it is unclear whether a primary barrier for plasma filtration occurs from the molecular pores of SD proteins such as nephrin, or due to factors such as negative charge repulsion and/or GBM gel permeability [25,118]. It also remains an important question whether nephrin is indeed a molecular spring, as nephrin shares homology with other elastic immunoglobulin proteins [38], and FN-III domains have the ability to unfold [110]. In combination with these suggested functions, it has been established that nephrin’s extracellular region can mediate interaction with other SD proteins (e.g., Crumbs2, NEPH1) [39,119], ligands (e.g., Gal-1) [120], and growth factors (e.g., HGF) [19], allowing for modular activity at nephrin’s cytoplasmic tail.

Despite being only 154 amino acids in length, nephrin’s cytoplasmic region regulates essential interactions with a large portion of SD proteins and signal transducers. Highlighting this importance, a genetic mutation that truncates nephrin’s cytoplasmic region, Fin_minor_, leads to congenital nephrotic syndrome of the Finnish type and absence of SD formation [17,121]. One feature of this cytoplasmic tail is a C-terminal, non-canonical PDZ domain-binding motif, which controls processes like cell survival, cell polarity, cell–cell adhesion scaffolding, and nephrin membrane localization [83,98,122,123]. Nephrin is also SUMOylated primarily at K1100, which helps to stabilize its localization at the cell membrane [124]. Crucially, many binding partners are regulated through phosphorylated residues (Figure 2B). While one fifth of these residues are predicted phosphorylated sites [125], the confirmed and conserved phosphorylated residues can be subdivided into the threonines, T1120 and T1125, group A tyrosines, Y1114, Y1138, and Y1139, and group B tyrosines, Y1176, Y1193, and Y1217 [126]. The distinction between these tyrosine groups is the presence of Src Homology 2 (SH2)-binding YDxV motifs at each of the group B tyrosines [127], and when phosphorylated, they have particular affinity for certain interactors like the Nck family of adaptor proteins [95,128]. Nevertheless, due to the proximity of these residues, with each residue having many possible binding partners, elucidating nephrin signaling in specific contexts can be challenging. Therefore, to help understand this interplay, a summary of the described interactions to follow is given in Figure 2 and Table 1.

**Table 1:** List of established nephrin interactors via direct or unknown mechanism

Binding partner	Nephrin residues (human) that regulate binding	Function
14-3-3θ [129]	N/A	Immune signal inhibition
αII/βII spectrins [62]	N/A	Cell–cell junction regulation
α-actinin-4 [62]	N/A	Cell–cell junction regulation
kAE1 [130]	N/A	Cell–matrix signaling
aPKCι/λ/ζ [99,123,129,131]	N/A	Polarity, trafficking, immune regulation
Arf6 [132]	Phospho-dependent (undetermined site)	Lamellipodia and adhesion
βarrestin2 [133–136]	pT1120/pT1125/Y1193/Y1217	Nephrin endocytosis
C1-Ten [137]	Y1138	Nephrin dephosphorylation
c-Abl [138]	Phospho-dependent (undetermined site)	AngII-induced actin reorganization
Calcineurin-A [139]	N/A	Phosphatase control in injury
Cask [62]	N/A	Adhesion regulation and cross-talk
Caveolin-1 [140]	N/A	Nephrin endocytosis
CD2AP [141,142]	N/A	Cell survival signaling
CIN85/RukL [143,144]	N/A	Nephrin ubiquitination/endocytosis
Claudin-1 [85]	N/A	SD disruption in disease
Crk1/2 /L [59,145,146]	pY1114/pY1138/pY1176/pY1193/pY1217	Cell migration and adhesion signaling
Crumbs2 [119]	N/A	Slit diaphragm architecture
Dendrin [147]	N/A	Inhibition of apoptosis
Densin [148,149]	N/A	Polarity and cell–cell adhesion
EphrinB1 [150]	N/A	Stress response signaling/motility
FAK [59]	N/A	Adhesion cross-talk and motility
Fyn [151,152]	pY1176/pY1193/pY1217	Nephrin phosphorylation
Gal-1 [120]	N/A	Glycan-binding and growth signaling
GIV/Girdin [153]	N/A	Nephrin phosphorylation/actin remodeling
HGF [19]	N/A	Nephrin phosphorylation/actin remodeling
ILK [77]	N/A	Cell–cell junction regulation
IQGAP1 [62,154,155]	N/A	Cell–cell junction regulation
IR-B [156]	N/A	mTORC1 activation
Lyn [157]	N/A	Nephrin phosphorylation
MAGI1/2 [62,83,158,159]	PDZ-binding motif	Adhesion activation/differentiation
Myo1c [160]	N/A	Nephrin vesicle trafficking
Nck1/2 [95]	pY1176/pY1193/pY1217	Actin organization/adhesion/cell signaling
NEPH1-3 [104,161,162]	N/A	Control of nephrin phosphorylation/actin remodeling
NM-IIA [103]	N/A	Nephrin vesicle trafficking
NMDAR1 [163]	N/A	Synaptic glutamate signaling
p75 [164]	N/A	Inhibition of apoptosis
p85 [97,165]	pY1114/pY1138	Cell survival signaling
p120 catenin [37]	N/A	Cell–cell adhesion
PACSIN2 [166]	N/A	Endocytosis and vesicle trafficking
Par3/6 [98,99,131,167]	PDZ-binding motif to Par3, but remains disputed [122,167]	Adhesion, polarity, and vesicle trafficking
P-cadherin [37]	N/A	Cell–cell adhesion
PICK1 [134]	N/A	Nephrin endocytosis
PKCα [134]	T1120/T1125	Nephrin phosphorylation/endocytosis
PLCγ1 [100]	pY1176	Calcium signaling
PlexinA_1_ [168, 169^168^,^169^]	N/A	Regulation of cell growth
Podocin [105,106,170]	pY1193	Lipid raft assembly/differentiation
PSD95 [163]	N/A	Cell–cell adhesion scaffolding
PTP1B [171]	pY1193/pY1217	Nephrin dephosphorylation
ROBO2 [172]	Likely YDxV motifs (via Nck)	Nephrin-actin signal inhibition
Septin7 [103,141]	N/A	Nephrin vesicle trafficking
ShcA [173]	pY1176/pY1193/pY1217	Nephrin endocytosis
SHP-1 [174]	pY1114/pY1138	Nephrin dephosphorylation
SHP-2 [19,175]	pY1138/pY1158	Nephrin phosphorylation regulation
SIRPα [176]	N/A	Nephrin dephosphorylation
Slo1_VEDEC_ [61]	N/A	Membrane potential regulation
SNAP23 [103]	N/A	Nephrin vesicle trafficking
SUMO1/2 [124]	K1100	Decreased nephrin turnover
TBC1D8B [177]	N/A	Nephrin vesicle trafficking
TRPC6 [60]	N/A (but Fyn-dependent)	Calcium signaling regulation
VAMP2 [102,103,141]	N/A	Vesicle trafficking
Vangl2 [178]	N/A	Cell polarity and directional migration
VEGFR2 [179]	Y1176/pY1193	VEGF-A-induced actin reorganization
Yes1 [151]	N/A	Nephrin phosphorylation regulation
ZO-1 [37]	N/A	Cell–cell adhesion

This list excludes any binding partners that were only identified using high-throughput screening techniques. Binding partners with known indirect binding mechanisms are also excluded. Proteins that have high likelihood of binding in specific regions (e.g., PDZ-containing proteins), but have absent/inconsistent evidence of such binding, are still noted as N/A under residues that control binding*.*

### Nephrin’s PDZ-binding motif and other phospho-independent signals

#### MAGI-1/2 scaffold protein interactions

Nephrin’s C-terminal PDZ-binding motif controls crucial signals that can occur independently of nephrin phosphorylation. One critical example is the direct binding between nephrin and MAGI-1’s third PDZ domain [122,158]. With MAGI-1, nephrin forms a tripartite complex with JAM4 to help scaffold the SD junction and maintain nephrin membrane localization [83,158]. Importantly, this complex was also shown to co-ordinate the activation of Rap1 [83], a small GTPase responsible for inside-out β1 integrin activation via binding to talin-1, highlighting one of several signaling axes that demonstrate cross-talk between nephrin and FAs [180,181]. However, MAGI-1’s effect on Rap1 was also dependent on calcium signaling [83], suggesting that this signaling axis might require multiple signal activations for its full effect. If nephrin co-operates with calcium in this way, it should be noted that MAGI-1 might be an important link to another nephrin interactor, Slo1/BKCa [61,182], a calcium-activated potassium efflux channel that assists in membrane repolarization and the regulation of cellular excitability [183]. In addition to MAGI-1, other PDZ-containing proteins at the SD interact with nephrin (e.g., MAGI-2, ZO-1, CASK, PSD95, Par3, and NMDAR1) [37,98,163,184], although inconsistencies in the required binding regions have been observed, and the non-canonical nature of nephrin’s PDZ-binding motif might require complex mechanisms and additional structural elements for these interactions [98,122,184,185].

MAGI-2 is one such example, which requires its third to fifth PDZ domains and a longer portion of nephrin’s C-terminal tail for effective interaction [184,185]. In contrast with MAGI-1, MAGI-2 is essential for SD formation and can control actin organization, adhesion signaling, cell survival, SD component trafficking, and forms complexes with numerous proteins (e.g. RapGEF2, IQGAP1, CASK, αII/βII spectrins, α-actinin-4, dendrin, and CD2AP) [62,83,159,185–187]. Among these interactions, a nephrin-MAGI-2-RapGEF2-Rap1 signaling axis could be required for continuous SD-FA crosstalk, as podocyte-specific deletion of MAGI-2 or RapGEF2 leads to rapid glomerulosclerosis [186]. Another important function might stem from MAGI-2’s ability to undergo liquid-liquid phase separation in complex with dendrin and CD2AP [185]. These condensates efficiently retain their SD localization, where CD2AP likely helps connect nephrin to actin regulators (e.g. cortactin and capping protein) and helps promote p85/PI3K/Akt survival signals [96,188]. This retained dendrin membrane localization could also prevent dendrin nuclear import, which leads to altered FA structures, activated JNK, and increased apoptotic signaling [147,189]. In this way, nephrin localization to the SD alone, independent of its phosphorylation status, is sufficient to maintain signals for FA crosstalk, actin tethering, and survival signaling.

#### Polarity regulation

In a similar manner, nephrin membrane localization can elicit polarity signaling through its interaction with the PDZ-containing Par3/Par6/aPKC complex [98,129]. One effect of this interaction is the inhibition of NF-κB signaling, where membrane-bound nephrin sequesters 14-3-3θ and aPKCζ [129]. However, the necessity of this signal axis remains uncertain, as aPKCζ-KO mice have no reported renal phenotypes [190]. Instead, essential polarity signals appear to be carried out through Par3/6 and aPKCλ/ι complexes [98,99,123]. Specifically, introducing a dominant negative variant of aPKCλ, aPKCλ inhibition, or aPKCλ/ι KO *in vivo* leads to rapid proteinuria, altered SD protein surface localization, FP effacement, and glomerular injury that resembles FSGS [98,99,131,167]. Notably, these complexes via Par6 are known to recruit cdc42 [191], an essential Rho GTPase for podocyte development and function [192]. Therefore, their interaction with nephrin might help orient crucial filopodia growth and actin reorganization, which is supported by aPKC localizing to filopodial tips in podocytes [99]. Further, it has been hypothesized that nephrin’s interaction with the cdc42 activator, IQGAP, could aid in activating this Par6-localized cdc42 [62,98,193]. Alternatively, the primary GEF and GAP of cdc42 activity in podocytes are β-PIX/ARHGEF7 and cdGAP/ARHGAP31, respectively [194,195]. As these GEFs/GAPs also have important roles in FAs, filopodia assembly could require shared cdc42 regulation between the SD and FAs [194–196], though the spatiotemporal details of this activation are yet to be elucidated.

#### Vesicle trafficking

As an additional role, nephrin’s most C-terminal residues could control aspects of vesicle and membrane trafficking, although it remains unclear whether the PDZ-binding motif, independently of nephrin phosphorylation, can elicit this action. Specifically, nephrin’s C-terminus binds VAMP2 in a complex with syntaxin-4, SNAP23, and NM-IIA, which have shown crucial functions in the insulin-responsive exocytosis of GLUT1 and GLUT4 storage vesicles (GSVs) [102,103,141]. Importantly, the activity of NM-IIa for this exocytosis is inhibited when nephrin is in complex with CD2AP and septin7 (with possible contribution of septins 9 and 11 for this inhibition as well) [103,141]. This finding might provide an important clue regarding podocyte adaptability: nephrin’s interaction with CD2AP at the SD might regularly inhibit GSV exocytosis (and therefore increased glucose intake) to this area, while allowing up-regulation of this action when the integrity of the SD is disrupted. Interestingly, CD2AP controls multiple other mechanisms of nephrin endocytosis, as it prevents CIN85-mediated nephrin ubiquitination and shares interactions with dynamin, synaptojanin 1, and endophilin [144,197].

Finally, other mechanisms of vesicle trafficking might occur independent of nephrin phosphorylation, such as myo1c, caveolin-1, IQGAP1, PACSIN2, and TBC1D8B, but their mode of interaction remains uncharacterized [140,160,166,177,197–199]. For Caveolin-1, IQGAP1, and PACSIN2, they each promote increased nephrin internalization upon diabetic conditions, suggesting a tentative requirement of altered phosphorylation for their interaction, although PACSIN2’s binding appears to be dependent on palmitate treatment as well [166,198,199]. In the case of TBC1D8B, a Rab11 GAP, the N-terminal half of nephrin’s intracellular tail (residues 1084–1160) was sufficient for interaction, indicating a potential contribution from group A tyrosines or threonines 1120/1125 [177]. In this study, TBC1D8B and Rab11 co-ordinated the proportion of nephrin recycling to autophagosome maturation, which highlights a potential role for nephrin in adaptive autophagic responses that should be further explored. Nevertheless, the exact influence of nephrin phosphorylation on these processes can be challenging to discern, and as discussed in the subsequent section, nephrin has important mechanisms of vesicle trafficking that are dependent on its phosphorylation as well. Understanding the relationship between these phospho-independent and phospho-dependent interactions in the context of the SD could help explain how nephrin spatiotemporally controls specific adaptive responses.

### Phosphorylation-dependent signaling mechanisms

#### Regulation of nephrin’s phosphorylation status

For such dynamic responses, nephrin’s phosphorylation-dependent signals can be controlled through factors such as spatial orientation and kinase/phosphatase availability. One stimulus that induces phosphorylation of group A and B tyrosines is nephrin clustering [145,157]. If nephrin exists primarily in zipper conformation at the SD, this clustered conformation alone might induce its phosphorylation, which remains detected to a moderate degree in healthy conditions in mice [200]. Alternatively, clustering with NEPH1 [104], or binding to external factors like Gal-1 and HGF [19,120], can also induce nephrin phosphorylation. However, it is unclear how commonly this zipper conformation or NEPH1 interaction occurs [38], and it remains an important question whether additional stimuli, such as mechanical cues at the SD, are required to cluster nephrin molecules and co-ordinate internal signals. The primary kinase responsible for cluster-induced phosphorylation is the key Src family kinase (SFK), Fyn [151], although other SFKs such as Yes1, Lyn, and Src are capable of phosphorylating nephrin as well [151,157]. A positive feedback loop for this phosphorylation can then be promoted by subsequent recruitment of adaptor proteins like Nck1/2 and Shp2, which maintain Fyn activation and its localization to nephrin and the SD [152,175]. Inhibition, on the other hand, can be carried out by several phosphatases, including Shp1, PTP-PEST (indirectly), PTP1B, and C1-Ten, and many of these phosphatases have been associated with podocyte injury [137,171,174]. Nck adaptors can also bind PTP1B, PTP-PEST, and the Fyn regulator Fyb/ADAP [201–204]; thus, these adaptors might also aid in nephrin dephosphorylation under certain conditions (e.g., pathological tight junction re-formation at the SD) [203], but such roles have yet to be determined.

#### Interaction with podocin

One of the first demonstrated interactions stimulated by these contexts is podocin, which is enhanced by phosphorylation of the group B tyrosine Y1193 [135]. Podocin is an essential scaffolding protein that forms homo-oligomers and guides lipid raft formation at the SD [105]. Nephrin trafficking to these lipid rafts is controlled by podocin, as the R138Q podocin mutant leads to nephrin retention at the ER [133,170]. Likewise, podocin helps retain nephrin in lipid rafts, slowing nephrin’s diffusion from these microdomains [133]. Nephrin’s direct binding to podocin can also affect signal transduction, including activated stress responses such as JNK, p38, and AP-1 [106]. Although this signaling can be facilitated by pY1193-dependent interaction with podocin, nephrin might indirectly bind podocin through common phospho-independent binding partners like CD2AP, NEPH1, and TRPC6 [106,205,206]. Thus, it remains unclear how much the direct nephrin-podocin interaction via pY1193 is responsible for modulating these stress responses, or whether indirect mechanisms also contribute to nephrin–podocin signaling. Considering that these two proteins are among the most abundant at the SD, their mechanisms driving these stress responses should be further investigated.

#### Akt and survival signaling

PI3K/Akt-mediated signal transduction is another important pathway downstream of nephrin phosphorylation. Phosphorylation of the group A tyrosines Y1114 and Y1138 can recruit the p85 subunit of PI3K, and in concert with podocin and CD2AP, lead to PIP_3_ production and Akt activation [97,165]. Reported effects of this activation include activated Rac1 and cofilin-1, lamellipodia formation, GSK-3β inactivation, and prevention from anoikis [96,165]. However, it should be noted that podocytes *in vivo* primarily express the Akt2 paralog [207,208], and its activation has been associated with distinct signaling from other Akt paralogs, demonstrating a decrease in Rac1 rather than an increase [207]. Loss of Akt2 in podocytes impairs podocyte survival and morphology [207]; thus, if nephrin phosphorylation can be stimulated at times of compromised adhesion, Akt2’s effects against anoikis might be important for ensuring podocytes survive after FP detachment, maintaining their ability to reverse the acute injury and regenerate FPs. Additionally, as a central kinase in many cellular processes, Akt activations might allow direct or indirect cross-talk with FA and matrix components (e.g., the expression of β1 integrin, FAK, palladin, and fibronectin) [209–212]. Further research is needed into this signaling axis, and the absence of specific antibodies against nephrin’s group A tyrosines has made it challenging to determine the contexts that activate this pathway.

#### Calcium signaling

Next, there are several mechanisms for nephrin to regulate intracellular calcium cascades. As one manner of eliciting this function, nephrin phosphorylation at Y1193 stimulates its interaction with PLCγ1, subsequently leading to diacylglycerol (DAG)/IP_3_ generation and the release of ER calcium stores [100]. Alternative to PLCγ1, nephrin’s phosphorylated group B tyrosines can bind the Nck adaptor paralog Nck2, which binds another important PLC family member in podocytes, PLCε1 [213]. This interaction was initially suggested to have roles in cell migration, but it remains to be determined if Nck2 and PLCε1 have similar functions in calcium signaling as PLCγ1. Notably, both nephrin and PLCγ1 also interact with TRPC6, which has roles in extracellular calcium influx upon mechanical stimuli and osmotic stress [48]. In contrast with PLCγ1, nephrin binds TRPC6 regardless of its phosphorylation at Y1193 and inhibits TRPC6’s localization to the plasma membrane [60]. This feature might have an additional role in stress responses, as nephrin internalization or downregulation could trigger TRPC6 localization to the plasma membrane, leading to increased mechanosensitive calcium influx. Within the podocyte microenvironment, this mechanosensitivity could be vital for rapid calcium influx and signaling responses during periods of increased shear stress, but could also be pathological if it remains upregulated. Thus, there is slight evidence suggesting nephrin binding partners provide further regulation of these calcium-dependent effects. As an example, nephrin binds the VEDEC splice variant of the calcium-sensitive potassium channel Slo1 [61], which could aid in the repolarization of membrane potentials following increases in calcium.

In these ways, nephrin probably employs strict control of calcium to regulate the potent downstream effects on podocyte function. Among the many consequences of cytosolic calcium entry, calcium and DAG-activated GEFs (CalDAG-GEFs) might promote Rap1-mediated integrin activation [214], especially as Rap1-dependence on calcium has been previously shown in podocytes [83]. In contrast, the calcium-dependent protease calpain has been shown to cleave FA components such as talin-1 and FAK [215,216]. Thus, transient calcium signals could be helpful for FA activation and remodeling, whereas prolonged calcium activation might break down FA components and decrease adhesion. In agreement with this effect, prolonged calcium signaling might stimulate calcineurin, NFAT, and uPAR expression, where uPAR’s activation of β3 integrin has been associated with FSGS [217]. Another important effect of calcium is actomyosin contractility [218], so it is possible that any calcium-dependent force transmission can be sensed by nearby mechanosensitive FAs. This calcium-mediated effect on contractility could help explain nephrin’s ability to induce actomyosin activity, force transmission, and FA activation [58]. With shorter durations of contractility, this force might reinforce existing adhesions [219], but longer durations of contractility might lead to larger rearrangements of podocyte structure. Indeed, sarcomeric contractile structures are observed in several models of podocyte injury [28], although the development of these structures likely involves many factors in addition to calcium. Nevertheless, careful control of nephrin phosphorylation and its co-regulators (e.g., Slo1) might provide tight regulation of calcium, allowing for rapid adaptive functions while preventing long-term pathological changes.

#### Phosphorylation-mediated endocytosis

One way of controlling such nephrin activity is its use of phosphorylation to regulate endocytosis and turnover. For this process, nephrin has two main mechanisms of internalization: clathrin-mediated endocytosis (CME) and raft-mediated endocytosis (RME) [133]. The CME pathway can be initially triggered by the phosphorylation of threonines T1120 and T1125 by the kinase PKCα and its guiding scaffold PICK1 [134]. Then, if nephrin is dephosphorylated at Y1193, the clathrin adaptor β-arrestin2 can bind nephrin and signal rapid internalization [134,135]. Clathrin-dependent nephrin internalization has also been shown following its interaction with developmental/polarity membrane receptors like Notch1 and Vangl2, although threonine phosphorylation by PKCα was not required for Vangl2’s effect toward nephrin [178,220]. However, PKCα up-regulation occurs following diabetic podocyte injury [221], and it remains possible that known regulators of nephrin endocytosis associated with diabetic conditions (e.g., IQGAP1, PACSIN2) might be facilitated by threonine phosphorylation [166,199]. Alternatively, nephrin can undergo RME, which is stimulated by group B tyrosine phosphorylation and is characterized by a slower internalization of lipid raft components via adaptor proteins [133]. In this case, phosphorylation of nephrin’s three YDxV motifs allows multivalent binding of Nck adaptors, which recruit actin regulators and the vesicular scission proteins dynamin1/2 [222–224]. In a similar way, the ShcA adaptor can also bind these residues in pathological conditions and up-regulate nephrin endocytosis, although ShcA has interactions linking to clathrin as well [173]. Thus, there is likely a large cross-talk between these endocytic mechanisms, exemplified by phospho-nephrin’s potential link to CME via PLCγ1 interaction, DAG/calcium production, and their activation of PKCα [100,225]. Eventually, phosphorylated nephrin traffics to early endosomes, where it is either recycled back to the plasma membrane or targeted to lysosomes for degradation [133,173]. One condition where nephrin is targeted for degradation occurs in the presence of growth factors (e.g., FGF-4) and absence of CD2AP, where nephrin is then targeted for internalization via its ubiquitination by CIN85/RukL [144]. With these features of internalization, nephrin’s ability to regulate its own negative feedback could be an important step of controlling its adaptive responses. Further, due to similar endocytic machinery (e.g., calcium and PKCα) regulating other signaling centers such as FAs [226–228], it is possible that nephrin signaling also elicits certain forms of turnover at adjacent FP domains.

#### Signaling roles via Nck adaptors

Among nephrin’s most notable functions, group B tyrosine phosphorylation can induce many signals via the Nck family of adaptors, which consists of the two paralogs Nck1 and Nck2. Previously mentioned shared roles by these adaptors include dynamin recruitment for nephrin endocytosis and Fyn recruitment for enhanced nephrin phosphorylation, with Nck2 having a moderately larger Fyn-mediated effect [152,222]. Also previously mentioned was Nck2’s unique affinity for PLCε1, which was suggested to aid in cell migration [213]. However, the most characterized downstream function of nephrin-Nck1/2 interaction is their role in actin polymerization, where Nck1 or Nck2 can bind N-WASP and subsequently activate the Arp2/3 complex [95,229]. The Arp2/3 complex is a key actin-branching nucleator that initiates nascent actin filaments that polymerize at 70^o^ from mother actin filaments, driving processes like cell motility, altered cell/organelle shape, vesicle trafficking, and endocytosis [230,231]. Due to the multivalent binding residues/domains in the nephrin/Nck/N-WASP/Arp2/3 signaling axis, increased phosphorylation at the three group B tyrosines can provoke liquid–liquid phase-separated clusters that have extended membrane dwell time, enhanced actin polymerization, and contractile lateral movement [107,108,232,233]. Likewise, multivalent interactions play a role in nephrin’s internalization via Nck and dynamin, which has increased effects when more YDxV motifs are phosphorylated [222]. Separate from this pathway, Nck adaptors also trigger actin regulatory signals through their interaction and activation of p21-activated kinase 1/2 (PAK1/2) [234]. One of these signals is PAK1’s ability to bind filamin, which then signals SHIP2 and lamellipodin to promote PI(3,4)P2 generation and lamellipodia formation [235]. As lamellipodia resemble spreading during FP effacement, it was also shown that protamine sulfate or puromycin aminonucleoside injury *in vivo* were associated with increased activation of filamin and SHIP2 in glomeruli, suggesting that this Nck1/2-PAK1 axis is activated during certain contexts of podocyte stressors. Finally, as a broad signal that can affect overall podocyte structure, nephrin’s binding of Nck1/2 can inactivate the hippo pathway. When recruited by nephrin’s phosphorylated group B tyrosines, both Ncks can bind WT1-interacting protein (WTIP) to sequester the Yap inhibitor LATS1, allowing for Yap to enter the nucleus and promote transcriptional changes toward cell growth and adhesion reprogramming [236–238].

In line with these effects, Nck adaptors could be important tools in nephrin-FA cross-talk and podocyte adaptability. Among Nck1 and Nck2’s large set of shared and distinct binding partners [239,240], many of their interactors regulate both the SD and FAs and they might possess similar signaling pathways and feedback mechanisms. Along with their most established interactors having important roles at FAs (e.g., N-WASP) [241], other Nck binding partners that have roles at FAs include PAK, FAK, p130Cas, PINCH1, and α actinin-4/eplin/palladin actin-binding complexes [196,240,242–244]. Thus, it is reasonable that the proximity of nephrin and Nck to FAs could allow the coupling of certain downstream effects at both signaling regions, such as kinase activation, calcium influx, Rho GTPase activation, actin reorganization, lipid composition changes, and mechanical forces. Supporting this SD-FA cross-talk, nephrin phosphorylation is associated with the assembly of ILK, PINCH-1, and α-parvin (IPP) complexes, which are essential adhesion modules in podocytes [77,245]. While the mechanism of nephrin signaling to IPP complexes remains unknown, nephrin can complex with ILK and α actinin-4, and this binding might require PINCH-1 recruitment by the adaptor Nck2 [77,244]. Also specific to Nck2 is its selective role in binding PLCε1, which can activate Rap1 via GEF activity and increased cytosolic calcium [213,246]. This combination of direct Rap1 activation and calcium signaling might have enhanced effects on integrin activation, as nephrin-mediated activation of Rap1 was previously shown to be dependent on calcium [186]. Alternatively, Nck1 (although untested with Nck2) can link nephrin to a proline-rich region on Robo2, a core SD component and regulator of FP branching morphogenesis [94,172]. In contrast with other cross-talk mechanisms, upon binding to the guidance cue, Slit2, nephrin and Nck bind Robo2 to trigger actin depolymerization, NM-IIa inhibition via SRGAP1, and decreased expression of the FA components paxillin and vinculin [247]. Thus, Nck adaptors might help spatially modulate FA activity near activated nephrin and Slit2 paracrine/autocrine signals.

#### Crk1/2/L and additional adaptor-dependent signals

**Figure 3:**
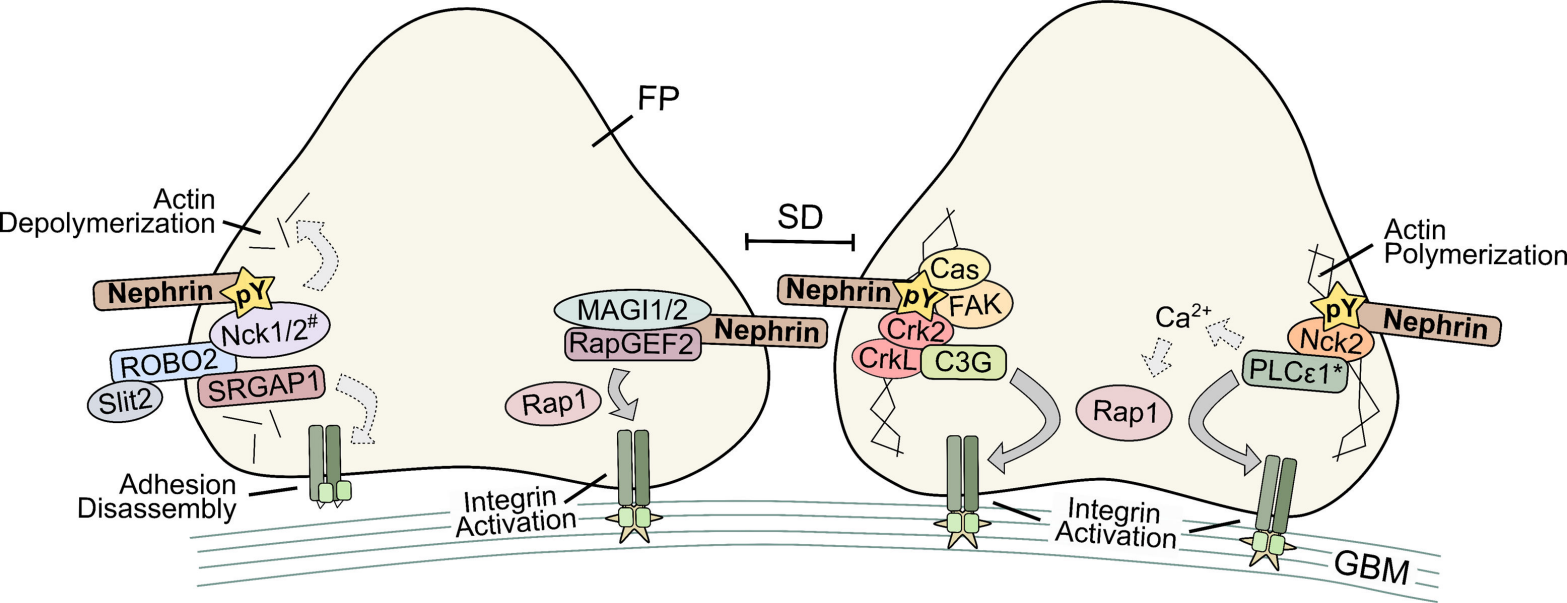
Mechanisms of nephrin–focal adhesion cross-talk. Illustration of four confirmed or proposed signaling axes downstream of nephrin that regulate integrin-containing focal adhesions. Direct signaling is indicated with arrows with a solid border, while indirect signals are shown with dashed border arrows with a lighter colour. Asterisk (*) indicates that calcium and Rap1 activation downstream of a nephrin-Nck2-PLCε1 axis has not yet been confirmed experimentally. Octothorpe (#) indicates that signaling between nephrin and ROBO2 has only been confirmed with Nck1 but potentially could be carried out via Nck2 as well. FP, foot process; GBM, glomerular basement membrane; pY, phosphotyrosine; SD, slit diaphragm.

Other major signal carriers have demonstrated nephrin’s cross-talk to FAs as well, including Crk adaptors, kinases, and small GTPases. Downstream of PI3K activation by phosphorylated group A tyrosines, p130Cas and FAK can bind nephrin, allowing for Crk1/2 and their paralog CrkL to bind to nephrin’s phosphorylated group B tyrosines [59,146]. Recruitment of Crk adaptors also appears to have a role in adaptive responses, as this pathway was associated with various injury models such as protamine sulfate and nephrotoxic serum (NTS). One key result of this signaling axis is FA modulation and lamellipodial activity [59], and importantly, it was later determined that the nephrin–Crk interaction recruits the Rap1 GEF, C3G, and increases β1 integrin activation [82,180]. With this additional pathway, nephrin has four aforementioned mechanisms that could lead to Rap1 and FA activation, with the other three mechanisms including 1) PDZ-binding motif, MAGI-2, and RapGEF2; 2) PLCε1 via Nck2; and 3) calcium CalDAG-GEFs, although the latter two mechanisms remain unconfirmed. For simplicity, an illustration of this Rap1 regulation and other nephrin–FA cross-talk mechanisms is given in Figure 3. Separately, nephrin’s phosphorylation can also elicit interaction with c-Abl, potentially via group B tyrosines as this process required c-Abl’s SH2 and SH3 domains [138,248]. This binding to c-Abl promotes cell migration and can inhibit c-Abl and SHIP2’s effect on angiotensin-induced deactivation of Akt [248]. As reduced activity of c-Abl was likely controlled by nephrin’s sequestration of c-Abl [138], it is possible this interaction also decreases c-Abl’s kinase activity at other regions like FAs [249]. Finally, nephrin–FA cross-talk has been established downstream of nephrin’s interaction with the small GTPase Arf6 [132]. Upon nephrin clustering and its phosphorylation, Arf6-bound nephrin contributed to Rac1 activation, lamellipodial protrusions, and FA turnover through β1 integrin endocytosis [132]. Such interaction was not important for steady-state podocyte functions due to Arf6 podocyte-specific KO mice having no structural or functional phenotypes. However, this interaction could be involved in adaptive responses to certain pathologies, as Arf6 podocyte KO mice had greater proteinuria following NTS and were resistant to protamine sulfate-induced effacement [132].

### Outlook and implications for nephrin

#### Exploring roles in adaptability

To better understand the role of nephrin phosphorylation *in vivo*, important insight has been gained from the generation of nephrin-Y3F mice, which have tyrosine-to-phenylalanine substitutions at each group B tyrosine. Surprisingly, with this loss of group B tyrosine phosphorylation *in vivo*, podocytes develop normally and have similar FP and SD structures at 1–2 months of age, indicating that these residues do not need to be continuously activated for steady-state kidney function or development [200]. Nevertheless, nephrin-Y3F mice quickly develop proteinuria and FP effacement, GBM protrusions, and capillary loop dilation as they age (starting from approximately 2–6 months of age) [200]. Furthermore, nephrin-Y3F mice have greater LPS-induced proteinuria, exacerbated hallmarks of diabetic nephropathy, and inhibited podocyte recovery following heparan sulfate administration after protamine sulfate injury [58,200]. In this way, nephrin group B phosphorylation was found to be essential to maintain podocyte integrity during stress and age-dependent changes.

Yet, phosphorylated nephrin can be observed in healthy mice, and this phosphorylation is associated with potent effects *in vitro* (e.g. lamellipodia/effacement, calcium influx, hippo inactivation, and endocytosis) [100,222,235,236], so the degree to which nephrin phosphorylation induces these powerful functions during healthy and pathological conditions *in vivo* remains unknown. If nephrin is continuously phosphorylated at multiple tyrosines, as is suggested by earlier models of the SD [111,115,157], these downstream effects would likely have negative consequences due to large-scale structural changes. Instead, it is possible that increased nephrin phosphorylation is an important driver of the transient effacement that was suggested to be a podocyte adaptive response [64], where nephrin phosphorylation first elicits effects like calcium signaling, force transmission, actin polymerization, and FA modulation, followed by its own negative feedback through raft-mediated endocytosis. However, to balance SD integrity with these adaptive responses, nephrin likely has sophisticated control of its phosphorylation-dependent effects, either through cellular context, interactor availability, and/or the valency of its phosphorylated residues.

#### Competitive phase induction

Regarding nephrin’s regulation via multivalency, nephrin has two confirmed signaling axes that involve liquid–liquid phase separation, those being nephrin-Nck-N-WASP and MAGI-2-dendrin-CD2AP [107,185], with the possibility of a third condensate consisting of p130Cas, FAK, and paxillin linked via group A tyrosines or Nck [59,250]. Because MAGI-2 binds via nephrin’s PDZ-binding motif, this mode of phase separation might occur independent of nephrin phosphorylation, allowing important functions during steady-state conditions. Indeed, MAGI-2, dendrin, and CD2AP, are among the top identified SD proteins in proteomic screens *in vivo* [93,94]. In contrast, phase separation with Nck and N-WASP is phosphorylation-dependent, particularly requiring hyperphosphorylation (i.e., simultaneous phosphorylation at each group B tyrosine) to allow condensate formation, powerful actin remodeling, contractile forces, and other downstream effects [107]. However, if nephrin has other high affinity interactors bound to it, or less phosphorylated tyrosines in proximity to one another, the strength of nephrin’s functions might be lower due to the absence of Nck-mediated phase separation.

Interestingly, the Nck-binding residues that provide phase separation are spatially close to the PDZ-binding motif (Figure 2B). Thus, it is important to determine whether MAGI-2 condensates can hinder phosphorylation of group B tyrosines, particularly Y1217, which is only 21 amino acids away from the PDZ-binding motif. This would indicate that nephrin has self-regulatory states of phase separation for different contexts, where MAGI-2 condensates might spatially inhibit Nck-mediated phase separation and vice versa. If steady states are associated with MAGI-2 complexes, it might require for the SD and these MAGI-2 condensates to be disrupted (potentially via shear/tension forces, nephrin autoantibody binding, protein glycation and misfolding, and lipotoxicity) to allow nephrin hyperphosphorylation, Nck-mediated phase separation, and downstream adaptive repair responses (e.g., force transmission, lamellipodia formation, nephrin and FA turnover, and hippo inactivation).

However, the effectiveness of this adaptive response would be dependent on the type and stage of injury. If injuries lead to the overactivation or underactivation of this nephrin activity, both these states might exacerbate pathogenesis from having maladaptive or absent repair responses. Furthermore, both these states might be involved during disease progression, with several observations showing increased nephrin phosphorylation in early disease, but decreased phosphorylation during later stages [87,251]. When nephrin is primarily dephosphorylated, it might correspond to states of up-regulated phosphatases (likely with threonine phosphorylation by PKC as well), which would lead to rapid nephrin internalization via clathrin-mediated endocytosis, and drastic changes such as loss of FP polarity, dendrin localization to the nucleus, and overall podocyte dedifferentiation [98,133,134,137,147,171,174,203]. Importantly, while most studies report nephrin dephosphorylation and decreased expression in disease, there remain many inconsistencies in detected nephrin levels following injury [87,100,173–175,200,252,253], and this variability could reflect whether nephrin is in a state of adaptability, maladaptability, or pathological down-regulation. Alternatively, observations of decreased nephrin during injury might reflect loss of podocyte density and detachment as well, so it is important to consider many factors when interpreting changes to nephrin from injury. Nevertheless, many pathological outcomes overlap with alterations in this signaling ability, supporting the idea that proper control of this signaling axis is key to mitigating injury.

#### Future topics and techniques to study nephrin

Although research efforts have improved our understanding of nephrin signaling, a crucial next step is to use the contexts of these signals to guide future clinical treatments. For this endeavour, one important question is whether there are ideal phosphorylation states for steady-state conditions and for FP repair, especially with how they relate to phase separation. For example, it is possible that nephrin dephosphorylation at single residues might be preferred to reduce Nck-mediated phase separation, as this might better control adaptive responses while still allowing other phospho-tyrosines to elicit Nck-mediated functions. If these ideal phosphorylation states are elucidated, it could suggest precise therapeutic targets for renal injury. As a tentative target, Y1217 is uniquely close to the PDZ-binding motif and might regulate nephrin’s phase separation with either MAGI-2-dendrin-CD2AP or Nck-N-WASP. If so, clinically targeting this residue via small molecules or kinases/phosphatases might improve podocyte adaptive responses to certain pathologies [107]. Such clinical interventions would have the added benefit of having less off-target effects, as nephrin has minimal expression outside podocytes (although pancreatic β cells, certain neuronal subtypes, Sertoli cells, and several other cell types might need important consideration) [164,254–259]. Nevertheless, these interventions require the investigation of nephrin’s three-dimensional structure *in vivo*, particularly when in interaction with other SD binding partners, and many challenges remain to develop drugs that target this region without disrupting SD integrity. As an alternative clinical approach, certain contexts might benefit from increasing nephrin expression, potentially via small molecules (e.g., PPARα agonists or dexamethasone) [260,261], engineered biomolecular approaches (e.g., small-activating RNA-tagged aptamers) [262], or nutrients (e.g., all-trans retinoic acid, 1,25-dihydroxyvitamin D_3_, or soy phytoestrogens) [261,263]. Finally, in cases of nephrin autoimmunity, immunosuppressive approaches are another clinical strategy that might lead to better renal outcomes, as previously demonstrated with the B-cell-depleting rituximab [112].

To guide these clinical challenges, many aspects of culturing podocytes and their microenvironment should be improved. While *in vitro* approaches featuring immortalized or primary podocytes have been essential for many insights of podocyte biology, this simplistic setting greatly differs from the complex *in vivo* environment. Specifically, cultured podocytes exist as a monolayer with minimal mechanical strain, altered substrate stiffness and composition, and various selection pressures, resulting in incomplete differentiation and limited expression of podocyte markers like nephrin and podocin [264,265]. Without these structures resembling the SD, it is challenging to determine which nephrin interactions have predominant roles during normal SD function or during SD disruption. Further, many of nephrin’s phosphorylation-dependent functions have been established using nephrin clustering systems. Such systems have greater nephrin membrane mobility *in vitro*, instead of being restrained to organized lipid rafts of the SD, potentially leading to distinct downstream effects than those *in vivo*. Thus, future work should investigate if mechanisms independent of clustering can also lead to nephrin phosphorylation. Nevertheless, nephrin clustering and its downstream functions are still a likely signaling event *in vivo*, although the exact instances that lead to this clustering (e.g. SD zipper formation, mechanical strain, clogs in the SD, and nephrin autoantibodies) should be further studied.

Fortunately, new approaches have been developed in the past decade to better study the native podocyte microenvironment, opening many research approaches for kidney development, drug screening, and disease modeling. Supplementing the existing animal models (e.g., mouse models, zebrafish pronephros, and the *Drosophila* nephrocyte) [266], current advances in *in vitro* models have integrated flow or stretch mechanisms with 2D stretch devices, organoids, or glomerulus-on-a-chip techniques to study complex glomerular cell states [267]. Although these approaches increasingly mimic native kidneys and show accurate filtration abilities [267–269], a major limitation remains mechanical strain. Across several leading techniques, one of the closest approaches mimicking the forces of the GFB cited shear stress of approximately 0.01 Pa [269,270], which is much lower than the 8 Pa being roughly estimated for *in vivo* shear forces [271]. Additionally, most chip devices are linear instead of the sharp curvature of the GFB and their shear forces often apply fluid flow apically rather than the perpendicular shear forces at the SD. As these forces can influence structure and how the shear/tension forces are distributed, future studies could try gradually applying perpendicular forces on podocytes on a suspended substrate to see whether this leads to the assembly of SD-like structures. Unfortunately, true reproduction of SD structures has not been successfully demonstrated *in vitro,* greatly limiting our understanding of nephrin signaling and podocyte biology in these models. Nevertheless, the rapid development of these new approaches, along with understanding the limitations of *in vitro* and *in vivo* studies, can still lead to major insights on podocyte function.

## Conclusion and significance

To summarize, great progress has been made to establish nephrin as a complex signaling molecule, but how all these signals co-operate with the complex SD setting remains an underappreciated area of study. While it is challenging to elucidate how nephrin’s diverse signaling modes can affect various podocyte structures, its opportune localization, expression, and potent functions make it important for further study. Although it may seem less essential to continue studying a molecule that has already had major discoveries regarding its function, new studies continue to highlight its importance, especially with many cases of nephrotic syndrome having an association with nephrin autoantibodies [18,112]. Therefore, a next step is to determine strategies that control nephrin’s function, either through its overall expression or careful control of its signaling modules. Such techniques modifying nephrin signaling might serve as an important route to greatly improving kidney health and our understanding of podocyte biology. In this way, just as nephrin sparked many initial discoveries about SD function, it is rightly being revisited for its large impacts on overall kidney health and disease.

## Abbreviations

FAs: focal adhesions
FP: foot process
GBM: glomerular basement membrane
GFB: glomerular filtration barrier
HSPGs: heparan sulfate proteoglycans
RLPs: ridge-like prominences
SD: slit diaphragm
